# Functional Profiling and Evolutionary Analysis of a Marine Microalgal Virus Pangenome

**DOI:** 10.3390/v15051116

**Published:** 2023-05-05

**Authors:** Briallen Lobb, Anson Shapter, Andrew C. Doxey, Jozef I. Nissimov

**Affiliations:** Department of Biology, University of Waterloo, 200 University Ave. West., Waterloo, ON N2L 3G1, Canada

**Keywords:** algal viruses, genomes, pangenomics, functional annotation, AlphaFold, Phycodnaviridae, Coccolithovirus, Chlorovirus, Prasinovirus

## Abstract

Phycodnaviridae are large double-stranded DNA viruses, which facilitate studies of host–virus interactions and co-evolution due to their prominence in algal infection and their role in the life cycle of algal blooms. However, the genomic interpretation of these viruses is hampered by a lack of functional information, stemming from the surprising number of hypothetical genes of unknown function. It is also unclear how many of these genes are widely shared within the clade. Using one of the most extensively characterized genera, Coccolithovirus, as a case study, we combined pangenome analysis, multiple functional annotation tools, AlphaFold structural modeling, and literature analysis to compare the core and accessory pangenome and assess support for novel functional predictions. We determined that the Coccolithovirus pangenome shares 30% of its genes with all 14 strains, making up the core. Notably, 34% of its genes were found in at most three strains. Core genes were enriched in early expression based on a transcriptomic dataset of Coccolithovirus EhV-201 algal infection, were more likely to be similar to host proteins than the non-core set, and were more likely to be involved in vital functions such as replication, recombination, and repair. In addition, we generated and collated annotations for the EhV representative EhV-86 from 12 different annotation sources, building up information for 142 previously hypothetical and putative membrane proteins. AlphaFold was further able to predict structures for 204 EhV-86 proteins with a modelling accuracy of good–high. These functional clues, combined with generated AlphaFold structures, provide a foundational framework for the future characterization of this model genus (and other giant viruses) and a further look into the evolution of the Coccolithovirus proteome.

## 1. Introduction

Viruses are the most abundant biological entities in aquatic environments, often substantially outnumbering their microbial hosts [1]. This abundance is also characterized by an enormous genetic diversity, with now thousands of new viruses being discovered every year due to advances in next-generation sequencing and the ability to directly assemble viral genomes from metagenomes [2]. In addition, viruses in the environment that infect other microbes, such as bacteria, archaea, and eukaryotic microalgae, also drive important ecological processes. They can contribute significantly to the demise of algal blooms in the ocean [3,4,5], and while doing so, help in the recirculation of important nutrients within tropic levels [6]. Moreover, viruses are intertwined with the evolution of their hosts and have the ability to contribute towards the flow of genes through horizontal gene transfer [7], as well as steal “auxiliary metabolic genes” (AMGs) that help them augment and/or supplement important host metabolic pathways [8].

One of the major virus families that infects marine and freshwater eukaryotic algae is Phycodnaviridae, which consists of large double-stranded DNA (dsDNA) viruses, with genomes of up to 560 kb in size [9,10]. Many of these viruses are experimental model systems that are used to study host–virus interactions and co-evolution in the context of ecological processes such as primary production, carbon flow, and nutrient cycling [9]. One commonly studied virus within the Phycodnaviridae family is Coccolithovirus, EhV-86. This virus is enveloped with a predominantly lytic cycle, replicates in the nucleus and assembles in the cytoplasm, and is released by budding or bursting the host cells [9,11,12]. At the centre of its ability to successfully infect and propagate is a de novo sphingolipid biosynthesis pathway [13], which is encoded on its genome and results in virally encoded glycosphingolipids [14], crucial virulence factors during infection [15].

Despite the existing knowledge on EhV-86 interactions with its globally distributed bloom-forming microalgal marine host, *Emiliania huxleyi*, our understanding of its molecular biology, evolution, and ecology is limited because functional annotations for most of the genes (86% for Coccolithovirus representative EhV-86) within completed and draft genomes is lacking. Indeed, many genomes can be characterized as containing viral genetic dark matter (vGDM), which refers to genes that are denoted as hypothetical or membrane proteins and are awaiting functional annotation. Given the intra-genus variability within different genera in the family, it is also not clear whether there are clusters of core genes unique to members of each genus, and if there are, what their phylogenetic ancestry is and what potential role they might play during infection.

Here, to shed light on the Coccolithovirus pangenome and vGDM, we performed a comprehensive analysis of viral genomes from the Coccolithovirus genus (i.e., one of the most studied Phycodnaviridae to date with global significance). We conducted a pangenome analysis of all 14 genomes within the Coccolithovirus clade and combined multiple protein annotation methods to predict the functions of Coccolithovirus hypothetical proteins. We also explored the potential biological significance of core genes that are expressed during infection using previously published transcriptome data obtained during *Emiliania huxleyi*–Coccolithovirus infection [16] and established a database of structural models for the majority of proteins within the Coccolithovirus genus. Ultimately, these analyses provide a framework for future studies on the evolution and function of the Coccolithovirus proteome.

## 2. Materials and Methods

### 2.1. Pangenome Analysis of the Genus Coccolithovirus and Comparison to Chloroviruses and Prasinoviruses

All available Coccolithovirus genomes were downloaded from the NCBI GenBank database (Table S1). PanX (pangenome analysis and exploration) v1.6.0 [17] was used to group gene sequences into gene clusters and to determine core and accessory genes for all Coccolithovirus genomes except for EhV-M1 (Table S2). For a gene to be considered core in our analysis, we used the default parameter of 100% so that a gene had to be present in all genomes of the clade. As EhV-M1 was recently sequenced during the course of this study [18], it was added into the pangenome through a BLASTN search of its coding sequences against the pangenome using BLAST+ v2.12.0, with each gene’s best match becoming its gene cluster (Table S2). Eight genes had no hits and were added to the pangenome as rare accessory genes. The SNP_whole_matrix.aln file generated in the panX tree-construction pipeline was subsequently used to generate a species tree for all Coccolithovirus genomes, with RAxML v8.2.12 (GTRGAMMA with 1000 runs). Using the gene cluster information from panX (with added EhV-M1), the micropan R package v2.1 [19] was used to apply a model of Heap’s law (heaps function) in order to estimate whether the pangenomes are “open” or “closed”, where a “closed” pangenome is defined by having an alpha value of >1, and an “open” one is defined as having an alpha value of <1. A Coccolithovirus rarefaction curve was also generated with the micropan package using the rarefaction function. Prokka v1.11 [20] with the kingdom viruses setting was used to re-predict genes in all Coccolithovirus strains. PanX and subsequent pangenome analyses, as described above, were used to create and assess a new pangenome for comparison purposes.

For the comparison of the Coccolithovirus pangenome to Chloroviruses and Prasinoviruses, we also downloaded their associated genomes from the NCBI (Table S3). The panX and subsequent Heap’s law analyses were conducted on each genus as described above for Coccolithoviruses.

### 2.2. Expanding the Coccolithovirus Annotation Profile

The expansion of the functional profile of the Coccolithovirus genus was carried out based on the genome of EhV-86, as it is the completed representative genome in the genus [12]. The annotation data for EhV-86 from JGI IMG/W v2.0 (“Emiliania huxleyi virus 86, isolate EhV86”) and Uniprot (including mapping to InterPro; “Emiliania huxleyi virus 86 (isolate United Kingdom/English Channel/1999)”) were downloaded on 27 October 2020. All other annotation methods were run on the EhV-86 (NC_007346) proteins from the NCBI protein database. The KofamKOALA webserver ver. 2020-10-04 [21] was used to search EhV-86 for KOfams against KEGG release 96.0. Pfam v33.1 [22] was searched with Pfamscan (available at ftp://ftp.ebi.ac.uk/pub/databases/Pfam/Tools/ and accessed on 4 November 2020) using HMMER v3.3 [23] with default settings. EhV-86 proteins were run through the PHYRE2 web server [24] in “normal” mode on 23 October 2020, with a threshold of 80% confidence and 30% alignment coverage to retrieve structural matches with putative functional relevance. EggNOG v2 (emapper v2.0.1b) [25] with the diamond setting was used to search the EggNOG database v5.0.0. In addition, hhblits v3.3.0 [26] was used to search the UniRef30_2020_06 database [27] (Uniclust; available here: http://wwwuser.gwdg.de/~compbiol/uniclust/2020_06/ (accessed on 4 November 2020)) with an E-value threshold of 0.001. Cluster names and organism breakdowns were taken from comparable UniRef50 and UniRef90 clusters. The remote homology PDB search results were taken from Mirzakhanyan and Gershon (2020) [28] (available here: https://sites.google.com/view/gershonlab-hhsearch-results/results (accessed on 4 November 2020)). Manually curated annotations of EhV-201 from Ku et al. [16] were mapped to EhV-86 using the pangenome data. Additionally, AlphaFold v2.1.1 [29] was run on each EhV-86 protein using the full set of databases as outlined by the tool, with the top-scoring models for each protein published on Dryad [30]. 25 proteins were too long to feasibly run and do not have results. Using the AlphaFold-derived structures, Foldseek [31] was run on EhV-86 proteins that had similar results from at least four sources (well supported) using the AlphaFold/UniProt50 v4, AlphaFold/Swiss-Prot v4, AlphaFold/Proteome v4, CATH50 4.3.0, GMGCL 2204, and the PDB100 2,201,222 databases.

### 2.3. Taxonomy Distribution of EhV-86 Protein Homologs

EhV-86 proteins were queried with a BLASTP search using the web interface for up to a maximum of 250 hits against the NCBI-nr database and an E-value threshold of 0.01 on 15 February 2021. ehv060, ehv192, ehv204, and ehv364 were long and/or repetitive sequences and were not included in this analysis. The genus, family, phylum, and super-kingdom levels from the taxonomy of each hit were examined at 80–100%, 40–80%, and 0–40% alignment identity. The most common (majority greater than one) lowest (genus prioritized first) taxonomic level in each category was identified for every gene. A hypergeometric test (phyper function in R v4.1.1 with lower.tail = false) was used to determine the enrichment of core or accessory fractions amongst sets of genes with common taxon matches.

### 2.4. Transcriptome Analysis

A gene expression dataset of a 24 h infection of *Emiliania huxleyi* CCMP2090 by EhV-201 from Ku et al. [16] was obtained from their Supplementary Materials and was mapped to the pangenome in our study. Core and accessory gene fractions were assessed for enrichment in the different phases of infection with a hypergeometric test using the phyper function (lower. tail = false) in R v4.1.1.

## 3. Results and Discussion

### 3.1. The Coccolithovirus Pangenome

**Figure 1:**
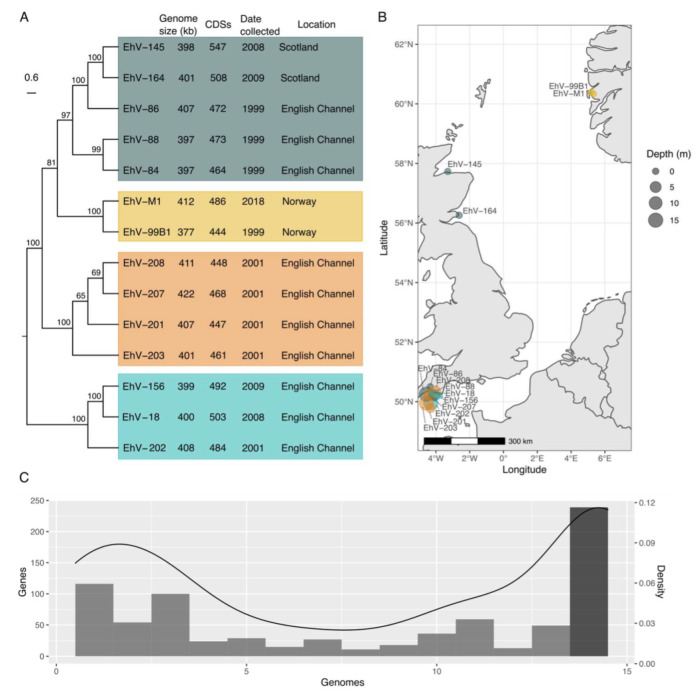
Phylogeny, geographical origin, and pangenome density of available Coccolithovirus genomes: (**A**) The phylogeny displayed here is a midpoint-rooted cladogram created with RAxML (GTRGAMMA) from the panX alignment data. The phylogeny with branch lengths displayed is in Figure S1. CDS numbers are from NCBI GenBank files and are slightly different than previously reported numbers in Nissimov et al. [12] from an alternate pipeline. (**B**) Approximate sampling locations around the coast of Great Britain and Norway. For exact GPS coordinates and depth, see Table S1. (**C**) Gene distribution across the 14 Coccolithovirus genomes, with the number of genes found in each genome. An aligned relative density estimate of the histogram is displayed on the alternative *y*-axis (right). The darker column represents genes that were shared across every Coccolithovirus strain in the pangenome. Re-annotated Coccolithoviruses (using Prokka) were also used to create a pangenome (displayed in Figure S2) with a very similar distribution (core genes changing from 30.25% to 34.50% of the pangenome).

We analyzed the currently available 14 Coccolithovirus genomes originating from different locations across the English Channel, Scotland, and Norway (Figure 1A,B and Table S1). In order to characterize the core versus accessory genes within the available Coccolithovirus genomes, we performed a pangenome analysis using the panX software [17]. A total of 790 genes were identified in the Coccolithovirus pangenome, 239 of which were identified as shared “core” genes present in all genomes (Figure 1C and Table S2). Across the remaining “accessory” gene fraction, there is a range in gene frequency from rare genes (e.g., 272 genes found in only 1–3 genomes) to more commonly occurring genes. It is important to note that, because almost all of the genomes are partially sequenced, in reality, some accessory genes may be more frequent throughout the clade. However, only 15 of the 272 rare gene clusters were found in EhV-86 (a fully sequenced representative), making it more likely that these are indeed rare accessory genes.

Based on a concatenated alignment of the single nucleotide polymorphisms (SNPs) from all single-copy core genes, phylogenomic analysis clustered these genomes into four subclades (Figure 1A), which is consistent with previous observations based on marker genes such as DNA polymerase and the major capsid protein [12,32,33]. The isolates from the English Channel fall into three different clades, with the Scottish isolates being the most similar to the EhV representative EhV-86, and the Norwegian isolates forming their own distinct grouping.

The presence and absence of genes across the pangenome lead to a clustering pattern almost identical to the SNP-based phylogeny (Figure S3), with the only difference being clustering patterns inside one of the main clades (EhV-201, EhV-203, EhV-207, and EhV-208). There are 114 clade-specific genes that help drive the overall clustering pattern, defined here as genes that are present in all clade members but absent in all other genomes. However, these genes only make up ~14% of the total pangenome. The most divergent clade (EhV-18, EhV-156, and EhV-202) has the most clade-specific genes, 86, which include a putative ribonucleoside diphosphate reductase, lectin, serine protease, polyubiquitin, and nucleic acid independent nucleoside triphosphatase proteins. The clade made up of Coccolithoviruses from Norway (EhV-99B1 and EhV-M1) only has seven clade-specific genes, including putative endonucleases and a putative transposase. The remaining two clades (EhV-86, EhV-84, EhV-88, EhV-145, and EhV-164; and EhV-201, EhV-203, EhV-207, and EhV-208) have three and eighteen clade-specific genes, respectively.

With increasing numbers of genomes within this clade, we observed a plateau in the number of identified orthologous genes (Figure S4). A Heap’s law analysis (alpha = 1.22) of these data estimated a “closed” pangenome, where newly sequenced genomes are unlikely to add significantly more new genes to the pangenome. A case in point is the newly sequenced EhV-M1 [18], which only added eight new genes to the pangenome and retained all the core genes previously established within Coccolithoviruses. An important caveat is that the available Coccolithoviruses have been isolated from similar regions (i.e., mainly coastal North Atlantic Ocean water), potentially leading to an underestimation of genetic diversity. Nevertheless, this same analysis performed without EhV-84, EhV-88, EhV-202, and EhV-203 (to better balance the isolation location and collection time within the analysis) still had an alpha greater than one (1.27), indicating a “closed” pangenome.

Although panX estimated an alpha value of 1.25 for Coccolithoviruses, the same analysis applied to another Phycodnaviridae genus, Chloroviruses (Table S3), resulted in an alpha of 0.52, indicating an “open” pangenome. This is supported by a recent pangenome analysis by Rodrigues et al. (2022) [34]. The number of gene clusters with an increasing number of genomes within this clade has not yet plateaued, probably because this clade is extremely multiphyletic. Most (2108/2780, ~76%) of the gene clusters in Chlorovirus genomes are present at low frequencies (one to three genomes), corresponding to a high percentage of rare accessory genes (Figure S5A) and a very small core genome (104/2780, ~3.7%).

Similarly, a Heap’s law analysis of Prasinovirus genomes resulted in an alpha value of 0.38, also indicative of an “open” pangenome (Table S3). Only 15/1262 gene clusters in Prasinoviruses were found in all genomes (Figure S5B). A very high proportion (1017 = ~81%) of Prasinovirus gene clusters are present at a low frequency (one to three genomes), corresponding to a high percentage of rare accessory genes within this group.

Interestingly, although the 14 Coccolithovirus genomes resulted in a “closed” pangenome, the “open” pangenomes detected for the Chloroviruses and Prasinoviruses suggest that these are genera with high genetic divergence. Chloroviruses, in particular, are multiphyletic [34,35]. Compared with EhVs, a substantially smaller number and proportion of genes in these two genera are core genes, with very few genes conserved throughout the group. Under the taxonomic structuring of these genera, host algal species are the differentiating factor between different taxonomic groups (i.e., all Coccolithoviruses are currently only known to infect the Coccolithophore species *Emiliania huxleyi*). Comparatively, Chloroviruses are known to infect species of *Chlorella variabilis*, *Micractinium conductrix*, and *Chlorella heliozoae* [34]. Prasinoviruses are known to infect a number of different species as well, including *Ostreococcus tauri*, *Micromonas pusilla*, and *Bathycoccus prasinos* [36]. Another aspect worth considering is that the hosts of Chloroviruses and Prasinoviruses can be found in both freshwater and marine environments [37,38,39], and currently sequenced viruses in these genera, for Prasinoviruses in particular, represent this diverse range of environments [37,40]. In contrast, the hosts of Coccolithoviruses are only found in marine environments, and compounding this, the currently sequenced Coccolithoviruses are from a very small number of geographic locations. It is therefore possible that the sequencing of additional Coccolithoviruses from a greater diversity of environments (gradient of brackish to saline) will provide new genomes that are distinct enough from the previously sequenced strains. This could potentially “shake up the current status quo” of the Coccolithovirus pangenome, reopen the pangenome, and establish distinct clades as in the other Phycodnaviridae we have analyzed here.

**Figure 2:**
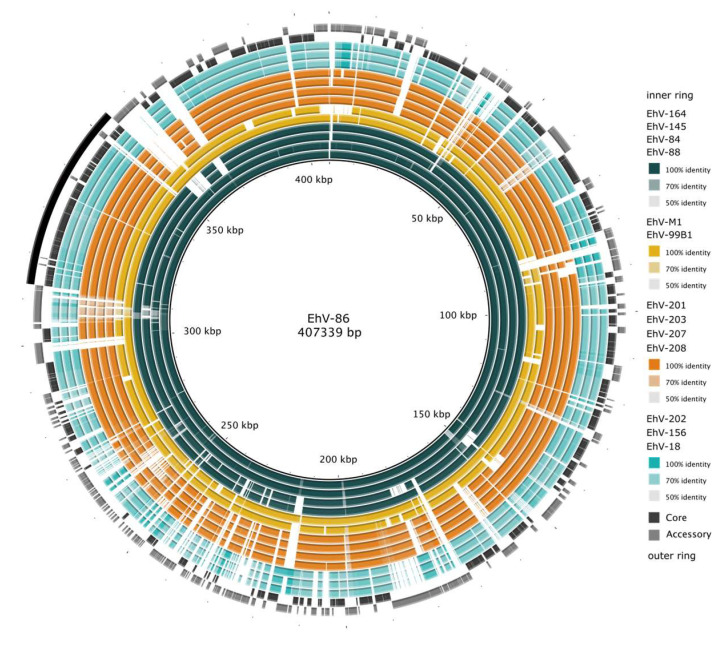
Genome comparison of Coccolithoviruses using EhV-86 as a representative. NC_007346 was used as the query against all Coccolithovirus genomes. The percent alignment identity across regions of EhV-86 to other Coccolithovirus strains is indicated based on colour intensity. Rings (EhV strains) are coloured and grouped by subclade. The black bar indicates a long region of interest with predominantly core genes. Generated with BLAST Ring Image Generator (BRIG) v0.95 [41]. This alignment shows the genetic content in the pangenome in relation to EhV-86. The genomes have different lengths and different numbers of genes (see Table S1). For an overview of the pangenome gene presence/absence, see Figure S2.

Next, we visualized genomic similarities among the 14 available Coccolithoviruses, by comparing them with the fully sequenced clade representative, EhV-86 (Figure 2). A BLAST ring image generator (BRIG) [41] plot shows that, as per the definition, core regions tend to correspond with well-conserved regions across the clade, while accessory regions tend to correspond with regions of deletions and with genetic variability. Of note is a large ~40 kb long region with predominantly core genes (49 core genes and 4 accessory genes) starting at 313, 210 bp and ending at 353, 998 bp (highlighted in Figure 2). This region consists of genes encoding the DnaJ domain-containing protein, CRAL-TRIO domain-containing protein, dUTP diphosphatase, putative DNA-directed RNA polymerase subunit, Lipocln_cytosolic_FA-bd_dom domain-containing protein, ribonuclease HII, putative protein kinase, A2L_zn_ribbon domain-containing protein, and putative fatty acid desaturase. The remainder of the genes within this region have been previously labelled as either “hypothetical proteins” or “putative membrane proteins”. Unannotated proteins are common throughout EhV-86 [12], with only 18% of its proteins having so far predicted products.

### 3.2. Expanding the Coccolithovirus Functional Profile

**Figure 3:**
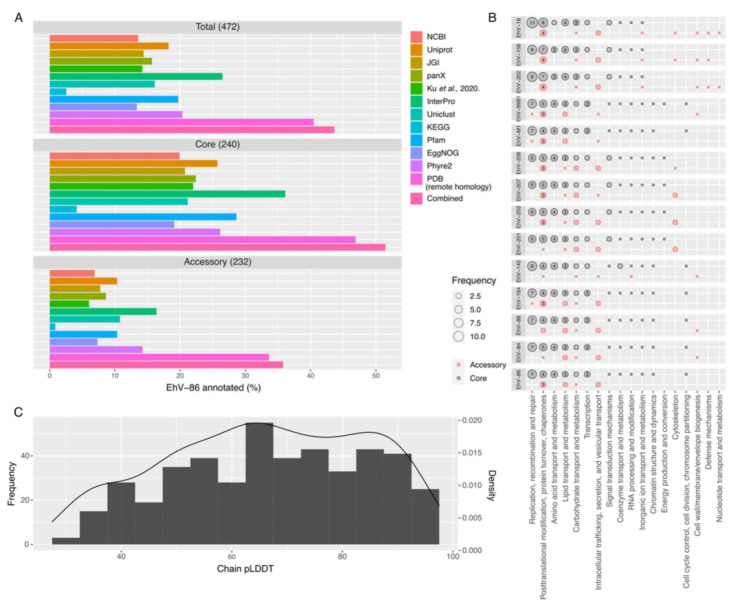
Annotation coverage and AlphaFold results for EhV-86: (**A**) All 12 annotation sources collected and/or generated for EhV-86. Any proteins called “hypothetical protein”, “putative membrane protein”, “uncharacterized protein”, or (in the case of EggNOG) not having any free text description were not included as “annotated”. Combined indicates all proteins with at least one annotation in any of the other categories. The Ku et al. [16] annotation set is a manual curation of EhV-201 annotations from other papers as well as BLAST searches against a variety of databases. The PDB (remote homology) annotations were retrieved from Mirzakhanyan and Gershon [28]. These data are included in Table S4. (**B**) Frequency of COG categories in Coccolithoviruses, divided into the core and accessory pangenome. Frequency here is the number of genes with the COG category in a genome. Counts less than 3 are not labelled. COG annotations derive from the EggNOG annotation database using emapper v2.0.1b. This method, likely due to genetic differences, does not always annotate every gene clustered together by panX in the same way (meaning that core genes do not always have equal counts of functorial categories across the figure). (**C**) The chain pLDDT scores for the best scoring model AlphaFold predicted for EhV-86 proteins. Chain pLDDT (predicted local distance difference test) scores closer to 100 show greater overall model confidence, being an average of the per-residue pLDDT values across the entire predicted structure.

To explore the current functional repertoire of the Coccolithovirus genus and the vGDM within it, we used the complete genome of EhV-86 and made an extensive comparison to major protein and annotation databases, as well as incorporating annotations from other publications (Figure 3A and Table S4). It is important to note that “annotations” (in our work and elsewhere) vary depending on the database, annotation method, and choice of bioinformatic parameters [42]. Some databases combine annotations from many sources and can have different approaches to providing functional information. Some methods have high stringency, while other methods are intended to be more sensitive and reach broader targets. However, the more functional clues that can be collected for a protein, the more information there is available to assign it a functional role with a higher degree of confidence.

We collected EhV-86 protein product names from NCBI, Uniprot, and JGI, which had been previously annotated using their own annotation pipelines. panX’s own annotation summary information, which compares the annotations from other genomes within the pangenome, was incorporated as well. The pangenome data also allowed us to map manually curated annotations of EhV-201 proteins from Ku et al. [16] to EhV-86. Additionally, we collected other public annotations against specific databases (e.g., InterPro from JGI’s pipeline [43] and a remote homology search against the PDB (Protein Data Bank) from Mirzakhanyan and Gershon [28]), and compared them with the up-to-date annotations we generated against other annotation databases (e.g., KEGG [21], Pfam [22], EggNOG [25], and Uniclust [27]). We also added another remote-homology-type method against the PDB that incorporates remote homology/threading-based structural modeling (Phyre2) [24] in order to find functional and structural commonalities between top model templates and other annotations.

Interestingly, Uniprot, and to a lesser extent JGI, have incorporated more database annotations into their own protein product names of EhV-86 than NCBI, leading to fewer hypothetical or “uncharacterized” proteins within their public annotations for EhV-86 (Figure 3A). NCBI has 64 (14%) named protein products for the EhV-86 proteome, while Uniprot has 86 (18%). Databases with domain annotations (e.g., InterPro and Pfam) annotate domains, which are smaller units of function. Because domains are often conserved regions within full-length proteins, even if a full-length protein has no annotations, domain matches may be found, leading to higher “annotation” coverage as seen here compared with other annotation sources. The remote homology search of PDB uses protein family models to look for distant evolutionary relationships, leading to high-level functional clues for proteins without annotations. Because this is the most sensitive approach, it has the highest annotation coverage (i.e., 40%; Figure 3A). While remote homology matches on their own provide insufficient information to give a hypothetical protein a product name, they can provide a direction to start pursuing and can provide support if cross-validated by other annotation methods.

Based on these 12 annotation sources, 44% (206) of the EhV-86 proteins were given a database match using at least one of these methods. This includes 142 of EhV-86′s vGDM (Table S4) that consisted of “hypothetical proteins” or “putative membrane proteins” (according to NCBI). Core genes were more likely to be annotated in general over accessory genes (52% versus 36%). This is possibly due to conserved, widespread functions involved in replication and DNA repair. In order to find well-supported putative protein products to assign to the vGDM within the core and accessory fractions, we compared the annotations across nine of the non-NCBI annotation sources (e.g., panX, Ku et al. (2020), InterPro, Uniclust, KEGG, Pfam, EggNOG, Phyre2, and PDB (remote homology)). The number of sources that provided similar information on a particular gene of an unknown function was used as a measure of annotation confidence (Table S5). We found functional information on 44 previously unannotated genes (and an additional 11 that expand on previous annotations), where at least 2 annotation sources agreed on a contained domain, protein family, and/or general structure, with 17 of these genes being well supported by at least 4 sources in consensus (Table 1).

Among these 17 genes, 5 core genes are worth considering further. The first two are predicted to encode RNA polymerase Rpb6, a homologue of which was previously found in an African swine fever virus [44], and a Poxvirus VLTF3, which was deemed as a trans-activator of late transcription in a vaccinia virus [45]. The third one is predicted to encode a telomere resolvase [46,47,48,49], a DNA breakage and reunion enzyme that was shown to be required in the maintenance of N15 bacteriophage lysogens as well as their lytic replication [50]. The last two are predicted to encode Heliorhodopsin, a recently discovered family of rhodopsins [51], which are photochemically active membrane-embedded proteins [52], previously detected in giant viruses such as those belonging to Phycodnaviridae [53]. Although it is difficult to speculate at this stage on the exact role that most of these play during Coccolithovirus infection, it has been previously hypothesized that virus-derived rhodopsins may indeed be involved in light sensing, which may impact the behaviour of their microalgal host [52]. A recent study also suggests more specifically that Coccolithoviruses may use heliorhodopsins to depolarize the host cell membrane, which consequently allows them to overcome host defenses and even prevent superinfection [54]. Furthermore, we can speculate that the potential presence of a telomere resolvase homologue may play a role in the chronic infection observed recently during Coccolithovirus infection, where it was shown that EhVs may exhibit a temperate lifestyle under certain conditions [55].

A closer look at the COG (clusters of orthologous groups of proteins) categories across the Coccolithovirus genus (Figure 3B) reveals that the core genes appear to be more likely involved in replication, recombination, and repair; post-translational modification, protein turnover, and chaperones; lipid transport and metabolism; transcription; and amino acid transport and metabolism. This makes sense given that these are all key functions in the life cycle of most viruses. In the case of genes involved in lipid transport and metabolism, it is well established that Coccolithoviruses encode their own set of sphingolipid biosynthesis genes, which are crucial for a successful infection [56,57] and in determining Coccolithovirus proliferation in the ocean [15,58].

The accessory genes of Coccolithoviruses on the other hand appear to be more likely involved in intracellular trafficking, secretion, and vesicular transport; cytoskeleton; and carbohydrate transport and metabolism (Figure 3B). Viruses closely associate with the cytoskeleton of eukaryotic cells and use it as a transport mechanism within the cell during infection [59]. It can be thus hypothesized that a virus that is less efficient in exploiting this system may thus be outcompeted by a virus that has some of these accessory genes. Indeed, during virus competition infection experiments, it was shown that although the competing Coccolithovirus strains EhV-86 and EhV-207 had similar adsorption kinetics, EhV-207 had a much shorter lytic period and numerically dominated during replication and release [60]. The competitive advantage of EhV-207 in the laboratory was eventually attributed to the efficiency with which this virus uses the host sphingolipid biosynthesis machinery [15], but it cannot be ruled out that there are other aspects that determine its success, such as superior use of intracellular trafficking and the cytoskeleton apparatus of the host. As it relates to carbohydrate transport and metabolism, it was previously shown that Chloroviruses encode many genes for proteins that are involved in the manipulation of carbohydrates, as well as other giant viruses [61,62]. The presence of these genes in only some Coccolithovirus strains may indeed explain the observed differential rate of polysaccharide production when some of them infect *E. huxleyi* [15].

While functional clues can help researchers piece together a more comprehensive picture of virus–host interactions, vast advancements have been made in the structure prediction field, providing yet another tool for understanding hypothetical proteins. In a first-of-its-kind analysis of giant virus proteomes, we predicted protein structures of EhV-86 proteins using AlphaFold (Figure 3C), an artificial-intelligence-based method [29], the results of which are now deposited in a publicly accessible database [30], which will add to the research community’s understanding of these viruses. Of the 408 hypothetical and putative membrane EhV-86 proteins, 153 of them received a predicted structure from AlphaFold with a chain pLDDT (the position-specific predicted local distance difference test score averaged across the entire amino acid chain) greater than 70 (Figure 3C), indicative of good–high model accuracy. Seventy-four of these proteins had no other hits with the other annotation methods examined in this study. Even with a chain pLDDT of 70 or more, structure prediction “success” can vary in part due to local differences across the structure. However, this is a promising start for the future characterization of vGDM. AlphaFold-generated structures for the well-supported EhV-86 proteins from Table 1 were further analyzed with the structure search tool Foldseek against several protein structure databases and confirmed the predicted annotations listed (Table 1 and Table S6). These protein structures can be a starting point for the functional discovery and further characterization of EhV-86 proteins and their orthologs in other viral genomes.

### 3.3. Taxonomy Distribution of EhV-86 Protein Homologues

**Figure 4:**
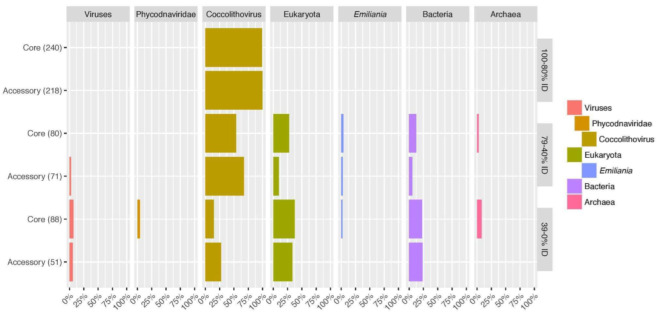
The majority taxonomy from EhV-86 BLASTP hits divided into different alignment percent identity ranges. The denominator for the percentages on the *x*-axis is indicated on the *y*-axis after the pangenome category (i.e., Core (240)). This is the number of EhV-86 proteins that had a majority taxonomy for that alignment percent identity range. The taxonomy in the legend is indented to indicate the taxonomic level. Only taxa present at greater than two percent within their category (*y*-axis) are displayed here. A full table of these data is available in Table S7.

An important question relates to the evolutionary and taxonomic origin of genes in Coccolithoviruses. It has been hypothesized that unique genes found in Coccolithoviruses may be evolutionarily derived from eukaryotic hosts or even potentially more distinct organisms across the tree of life [12]. To investigate this further, we used BLASTP to identify sequence matches to all EhV-86 proteins and then analyzed and visualized the taxonomic distributions of the top-scoring BLAST matches at different levels of sequence divergence (Figure 4 and Table S7; taxonomic distributions visualized on the genome in Figure S6). The genus, family, phylum, and super-kingdom levels from the taxonomy of each BLASTP hit were examined at 80–100% sequence identity (2420 sequence hits), 40–80% sequence identity (5904 sequence hits), and 0–40% sequence identity (14,571 sequence hits). These three sequence identity ranges capture different sets of proteins (i.e., close homologues to more distant sequences) based on their sequence divergence relative to the EhV-86 query. The majority taxon at each alignment identity range was then used to summarize the most common taxonomy of the protein’s BLASTP matches.

At high alignment percent identities (80–100%), the top BLASTP matches were almost all to other EhVs, represented here by Coccolithoviruses within the Phycodnaviridae family. Therefore, as expected, both the core and accessory genes of Ehv-86 have close homologues in at least one other Coccolithovirus strain. This supports the origin of these genes in ancestral Coccolithovirus strains. In the case of core genes, this ancestor was likely an early ancestor of all modern Coccolithoviruses, whereas accessory genes were more likely acquired in more recent ancestors of specific Coccolithovirus lineages. There are a few notable exceptions, however. These include core gene ehv367 (predicted as a metal binding protein), which had short, high percent identity alignments to bacterial proteins due to a shared SEC-C motif, and core gene ehv452 (involved in DNA binding), which had short, high percent identity alignments to eukaryotic proteins across a high-mobility group box domain.

Interestingly, at lower alignment percentage IDs (40–80%) associated with more distant homology relationships, we begin to see matches between core and accessory EhV-86 genes and genes in non-Coccolithovirus species (*p*-value 0.01, 69%). These species include various eukarya (encompassing the host organism, *Emiliania huxleyi*), bacteria, and archaea. As these genes are often widespread and well conserved (i.e., ribonucleases), their matches reflect more distant relationships to genes outside of viruses. We speculate that these matches reflect an evolutionary scenario in which an ancestral Coccolithovirus acquired many of its core and accessory genes from eukaryotic and bacterial/archaeal sources.

Notably, 5 proteins (ehv014—12-strain accessory longevity assurance (LAG1) family protein; ehv032—core putative membrane protein; ehv095—core putative membrane protein; ehv155—core putative membrane protein; and ehv424—4-strain accessory protein 3 source agreement glycosyltransferase) were mainly matched to proteins from *Emiliania*, the genus of the viruses’ known host, and 15 (11 core and 4 accessory) had at least one match specifically to *E. huxleyi* at the 40–80% identity range. Twelve proteins had the majority of their matches from a diverse range of taxa, including archaea (ehv105—core transcription factor S-II family protein and ehv167—core putative DNA-directed RNA polymerase subunit), viruses (ehv041—3-strain accessory putative endonuclease 4 source agreement GIY-YIG endonuclease domain-containing protein and ehv345—12-strain accessory hypothetical protein), *Ostreococcus* (green algae; ehv310—8-strain accessory putative membrane protein), and Planctomycetes (aquatic bacteria; ehv183—9-strain accessory labelled in JGI only as a serine/arginine repetitive matrix protein 2).

At low-alignment percent identities, most EhV-86 proteins mainly matched eukaryotic proteins (50 of the EhV-86 proteins). Furthermore, 32 mainly matched bacterial proteins, and 27 mainly matched proteins from Coccolithovirus. Viral proteins, archaeal proteins, phytoplankton (indicated by Haptista, *Emiliania*, and *Chrysochromulina*), *Chloropicon* (green algae), and *Rhodopirellula* (marine bacteria) were some of the most common taxonomic levels from the rest of the sequences.

Ultimately, these results suggest that the ancestor of modern-age Coccolithoviruses acquired genes from a range of sources, including eukaryotes. The distant homologues of many core and accessory genes may be derived from eukaryotic host and/or non-host distinct organisms, while a small subset of accessory genes have greater similarity to genes in other viruses. Some of the ancestrally acquired genes were universally conserved in all descendants becoming the core genome, whereas others have shown variable patterns of conservation or were acquired later in specific lineages (accessory genome). Most striking is how the Coccolithovirus genetic content seems to be an island in the database, with non-Coccolithoviral hits from other viruses only occurring in the sub-80 percent identity range but still rare until the alignment percent ID drops below 40%. This either implies that the ancestor of Coccolithoviruses underwent a substantial genetic shift away from other viral lineages or that other Phycodnaviridae and/or marine life are missing from our knowledge base.

### 3.4. Pangenome Expression Profile

**Figure 5:**
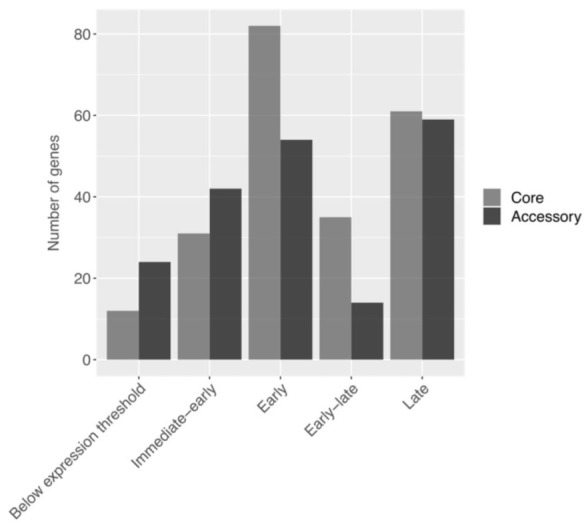
EhV-201 expression data from Ku et al. (2020) [16] broken up into core and accessory genes based on the pangenome analysis. The infection phase categories on the *x*-axis are from a hierarchical clustering analysis (MetaCell method using the k-nearest neighbor graph partitions) of the expression data performed by Ku et al. [16] looking at a 0–24 h post-infection time range of *E. huxleyi* CCMP2090 infected by EhV-201.

Finally, we wanted to explore the potential functional significance of the core and accessory genes that we identified in the Coccolithovirus pangenome. Using a transcriptome profile of a 24 h infection of *Emiliania huxleyi* CCMP2090 with EhV-201 previously developed by Ku et al. [16], we investigated how the core and accessory genes of the Coccolithovirus pangenome (Figure 5 and Table S5) separated across defined stages of infection (characterized by distinct promoter region motifs): “immediate-early”, “early”, “early-late”, and “late” phases (genes deemed by Ku et al. [16] as being in an “undetermined” or “early-undetermined” phase were omitted from our analysis).

Core genes were 1.5-fold and 2.1-fold enriched in the early and early-late phases of the infection, respectively, (*p*-values of 0.04 and 0.01), accounting for 60% and 71% of the genes associated with those phases, respectively. We thus suggest that most core Coccolithovirus genes play a pivotal role during the early stages of infection. Indeed, Ku et al. [16] concluded that the early-phase virus genes included almost all of the known genes for information processing and metabolism. The genes responsible for transcription and translation have been linked to core gene fractions, being essential for viral and cellular function [63].

Surprisingly, the genes deemed in our study as accessory genes were 1.7-fold enriched in the immediate-early phase (*p*-value 0.02, 58%) of infection. Ku et al. [16] demonstrated that these genes are also localized into two distinct sections on the EhV-201 genome, an aspect that may play a role in their rapid activation upon cell infection. Taken together, it can be hypothesized that the accessory genes that are involved in the immediate stage of infection and are localized together in a specific genomic region may have been acquired by the virus in a single horizontal gene transfer (HGT) event or several HGT events that encompassed a large proportion of these genes. Nevertheless, the fact that many of these accessory genes are not found across all Coccolithovirus isolates, yet they are clearly important upon infection, suggests that there may be multiple strategies for some of these viruses to accomplish successful infection, even in the absence of some of these genes.

In our analysis, accessory genes were also 2.0-fold enriched in the EhV-201 genes that ended up below the expression threshold during the *E. huxleyi* CCMP2090 infection experiment (*p*-value 0.01, 67%). Additionally, although the lack of detectable expression for some EhV genes during infection has been documented previously [13,64], no explanation has been provided yet on whether some of these genes are merely “genetic noise”, or whether they may play a role and get activated under certain conditions. Several studies illustrated that some Coccolithoviruses are “generalists”, infecting a wider array of *E. huxleyi* genotypes, whereas other ones are “specialists”, infecting a narrower range [15,65]. Unfortunately, a detailed side-by-side transcriptome analysis of “specialist” and “generalist” Coccolithovirus isolates is lacking.

Finally, our analysis showed no statistical differences between the enrichment of core vs. accessory genes in the late phase of infection, suggesting that both play an important role during the last phase of the virus replication cycle, in which virus DNA is packed into new virions, and the capsid is being assembled.

## 4. Conclusions

Our analysis revealed that although other members of the Phycodnaviridae family are extremely diverse and have open pangenomes, the available Coccolithovirus genomes are relatively conserved and are estimated to form a closed pangenome. However, this aspect may change when new viruses infecting other coccolithophore species are sequenced, either after isolation or by directly assembling new genomes from metagenomic studies. Within the Coccolithovirus pangenome, we identified several clusters of core and accessory genes that likely had different evolutionary trajectories, which are reflected in their different taxonomic distributions. Importantly, we found that in the context of infection, core and accessory genes may play different roles, as they are differentially enriched at various phases during host infection.

We also found that it is possible to illuminate the vGDM of giant viruses by using a multifaceted approach that incorporates data from a variety of functional annotation methods, protein databases, and manually curated annotations from prior studies. Building upon a confidence matrix that is based on the number of sources being in agreement with one another on a certain annotation, this approach can be a powerful tool. It expands our knowledge of the functional repertoire of this genus and, if applied in other studies, is likely to provide new information on vGDM contained within the genomes of other giant viruses. Finally, using AlphaFold, we constructed a first-of-its-kind database of predicted protein structures for a giant virus and made this publicly available, with the hope that it can be used by researchers interested in viral structural biology, an important component in fully understanding how a virus operates and what it targets in a host cell.

## Figures and Tables

**Table 1:** Hypothetical/uncharacterized genes with similar annotations from at least 4 sources. Bolded proteins are notable and further discussed. Annotations that overlap but are slightly different from the common annotation listed are included after the method with an asterisk in the final column. Every protein in this table was further analyzed with Foldseek [31], and the common match was checked for confirmation within the Foldseek results.

	Coccolithovirus Pangenome	Common Domain/Protein Family/Structural Matches for Non-Annotated Proteins	Number of Sources with Similar Hits	Sources with Similar Hits
ehv116	accessory (12 strains)	Fatty acid hydroxylase	8	InterPro, Pfam, PDB, Phyre2, EggNOG, Uniclust, Ku et al. [16], Foldseek
ehv138	accessory (12 strains)	Recombination endonuclease VII domain-containing protein	8	InterPro, PFAM, PDB, Phyre2, EggNOG, panX, Ku et al. [16], Foldseek
ehv088	accessory (12 strains)	Fatty acid hydroxylase	7	InterPro, Pfam, PDB, Phyre2, EggNOG, Uniclust, Foldseek
ehv411	core	Glycosyltransferase (GlcNAc)	7	InterPro, Pfam, PDB, EggNOG, Uniclust, Ku et al. [16], Foldseek
ehv452	core	HMG-box domain-containing protein (Uniclust annotated as signal recognition particle-docking protein FtsY)	7	InterPro, Pfam, PDB, Phyre2, EggNOG* (Chromatin structure and dynamics: DNA binding, bending), panX, Foldseek
ehv001	accessory (5 strains)	Recombination endonuclease VII domain-containing protein	6	InterPro, Pfam, PDB, Phyre2, Uniclust, Foldseek
ehv091	accessory (12 strains)	Zinc finger, C3HC4 RING-type	6	InterPro, Pfam, PDB, Phyre2, Uniclust, Foldseek
ehv403	core	**Poxvirus VLTF3**	6	InterPro, Pfam, EggNOG, Uniclust, Ku et al. [16], Foldseek
ehv463	accessory (9 strains)	NFACT protein RNA-binding domain-containing protein (can show up in fibronectin-/fibrinogen-binding proteins by similarity)	6	Pfam, PDB, Uniclust, panX, Ku et al. [16], Foldseek
ehv355	core	Phytanoyl-CoA dioxygenase domain-containing protein (Uniclust annotated as ricin B-type lectin domain-containing protein)	6	InterPro, Pfam, PDB, Phyre2, EggNOG* (acid phosphatase activity), Foldseek
ehv458	core	**RNA polymerase Rpb6**	6	InterPro, PDB*(RNA polymerase Rpb8), Phyre2, Uniclust* (DNA-directed RNA polymerase I II), panX, Foldseek
ehv058	core	Helicase C-like	5	InterPro, Pfam, PDB, EggNOG, Foldseek
ehv177	accessory (10 strains)	Zinc finger, RING-type	5	InterPro, PDB, Phyre2, Uniclust, Foldseek
ehv387	core	**Telomere resolvase**	5	InterPro, Pfam, PDB, Ku et al. [16], Foldseek
ehv367	core	SEC-C motif-containing protein	5	InterPro, Pfam, PDB, Phyre2* (low alignment coverage), Foldseek
ehv055	core	**Heliorhodopsin**	5	InterPro, Pfam, Phyre2, PDB* (rhodopsin), Foldseek
ehv078	core	**Heliorhodopsin**	5	InterPro, Pfam, Phyre2, PDB* (rhodopsin), Foldseek

## Data Availability

AlphaFold structures generated for EhV-86 proteins are published on Dryad [30]. All annotations are available in Tables S4–S6.

## Supplementary Materials

The following supporting information can be downloaded at: https://www.mdpi.com/article/10.3390/v15051116/s1, Figure S1: Coccolithovirus phylogenetic tree with branch lengths visualized.; Figure S2: Alternative gene distribution across the 14 Coccolithovirus genomes based on a Prokka re-annotation of the Coccolithovirus strains.; Figure S3: Clade specific genes within the Coccolithovirus pangenome.; Figure S4: Rarefaction curve of Coccolithovirus strains.; Figure S5: Pangenome distributions for Chloroviruses and Prasinoviruses.; Figure S6: The majority taxonomy of EhV-86 BLASTP hits, divided into three alignment percent identity ranges.; Table S1: Currently sequenced Coccolithoviruses and applicable metadata.; Table S2: PanX Coccolithovirus pangenome results.; Table S3: Prasinoviruses and Chloroviruses assessed for pangenome stability.; Table S4: All annotation results and collated annotation data for EhV-86.; Table S5: Annotation summary showing already established database protein product names and annotations with multiple source agreement that add to or expand on EhV-86’s annotations.; Table S6. Top 15 Foldseek matches of EhV-86 well-supported proteins sorted by probability and then E-value.; Table S7: Majority taxa of EhV-86 blastp hits against nr divided into three alignment percent identity ranges.
