# Supplementary material for: Functional Profiling and Evolutionary Analysis of a Marine Microalgal Virus Pangenome
Source: Viruses. 2023 May 5;15(5):1116. doi: 10.3390/v15051116 (PMC10222054; doi:10.3390/v15051116)
Supplement: Supplementary file 1 [file viruses-15-01116-s001.zip › viruses-2384873-supplementary.pdf]

## Supplementary Figures

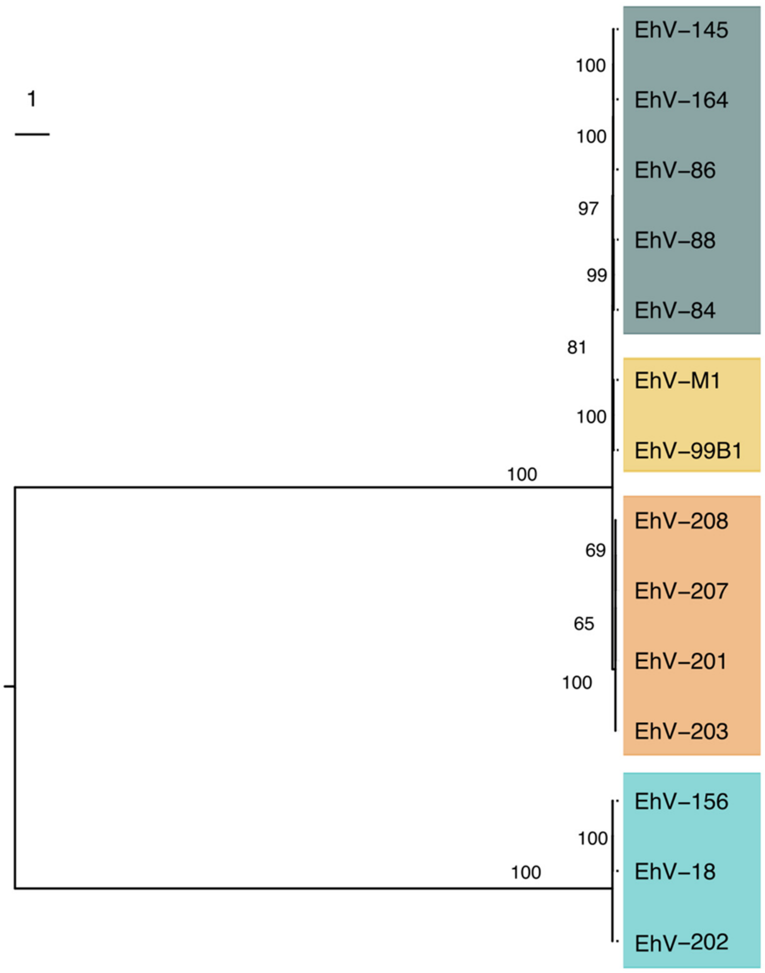


**Figure S1**. Coccolithovirus phylogenetic tree with branch lengths visualized. Midpoint-rooted tree was created with RAxML (GTRGAMMA) from a panX-generated SNP alignment. Cladogram is shown in **Figure 1A**.

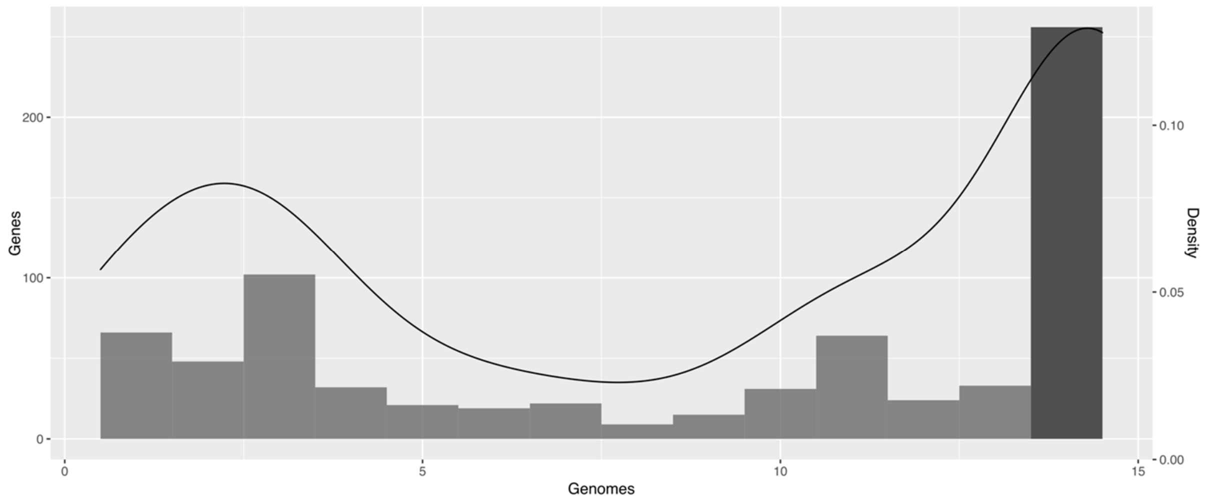


**Figure S2**. Alternative gene distribution across the 14 Coccolithovirus genomes based on a Prokka re-annotation of the Coccolithovirus strains. An aligned relative density estimate of the histogram is displayed on the alternative y-axis (right). The darker column is the core genome that is shared in all strains. The total genes in the pangenome decreased to 742 from 790 when compared to the original GenBank files. The core genes increased from 239 (30.25%) to 256 (34.50%). The rare genes (present in 1–3 genomes) decreased from 272 (34.43%) to 216 (29.11%). A Heap's law analysis still estimated a "closed" pangenome (alpha value greater than one), with the alpha increasing from 1.22 to 1.37. When EhV-84, EhV-88, EhV-202, and EhV-203 were removed to better balance the isolation location and collection time within the analysis, the pangenome was still estimated to be closed, with the alpha increasing from 1.27 to 1.42. Clade-specific genes increased from 114 (14.43%) to 130 (17.52%).

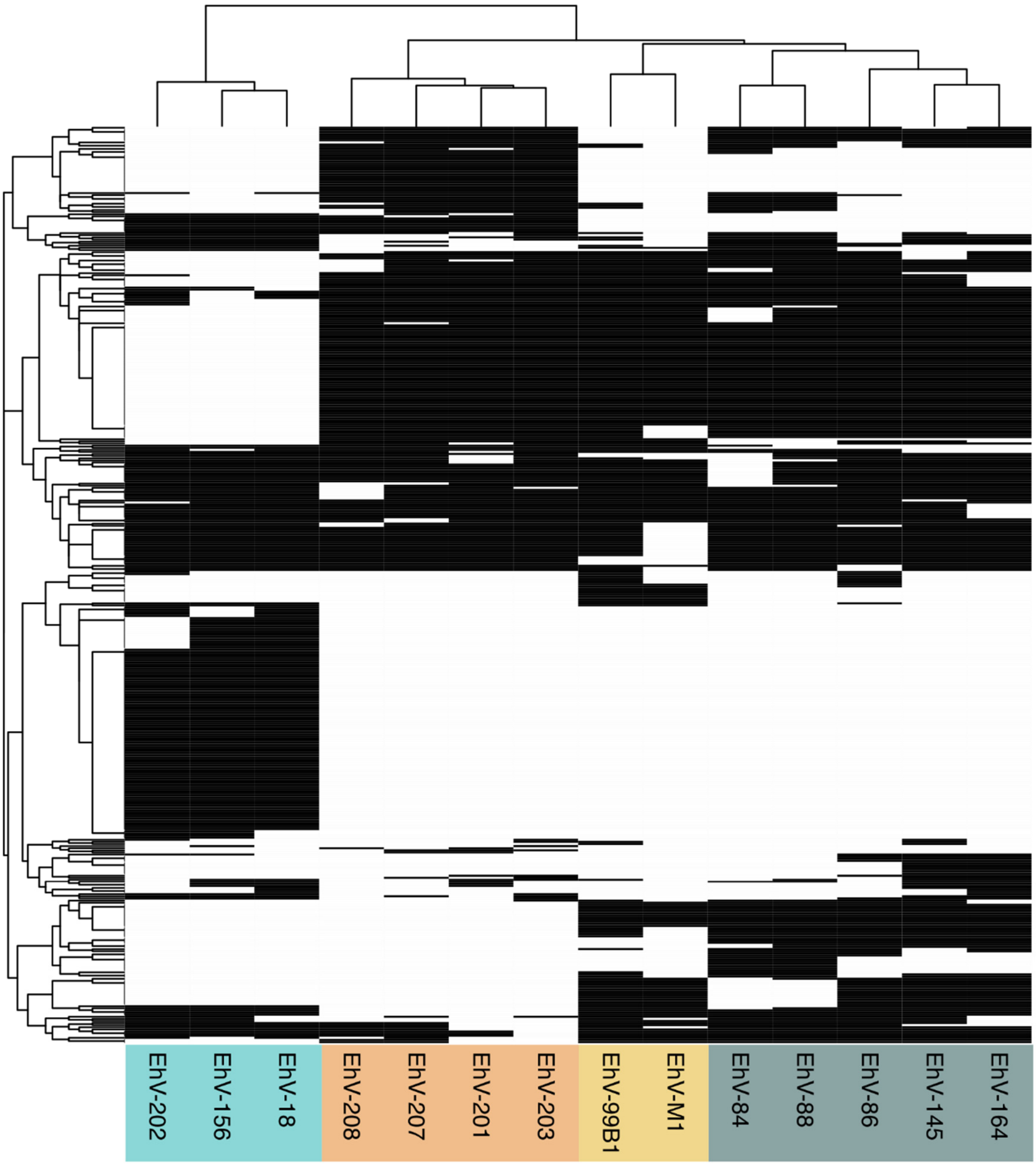


**Figure S3**. Clade-specific genes within the Coccolithovirus pangenome. The presence (in black) and absence (in white) of genes that are only present in 2-13 genomes (not core genes or genes only found in one genome) are displayed here.

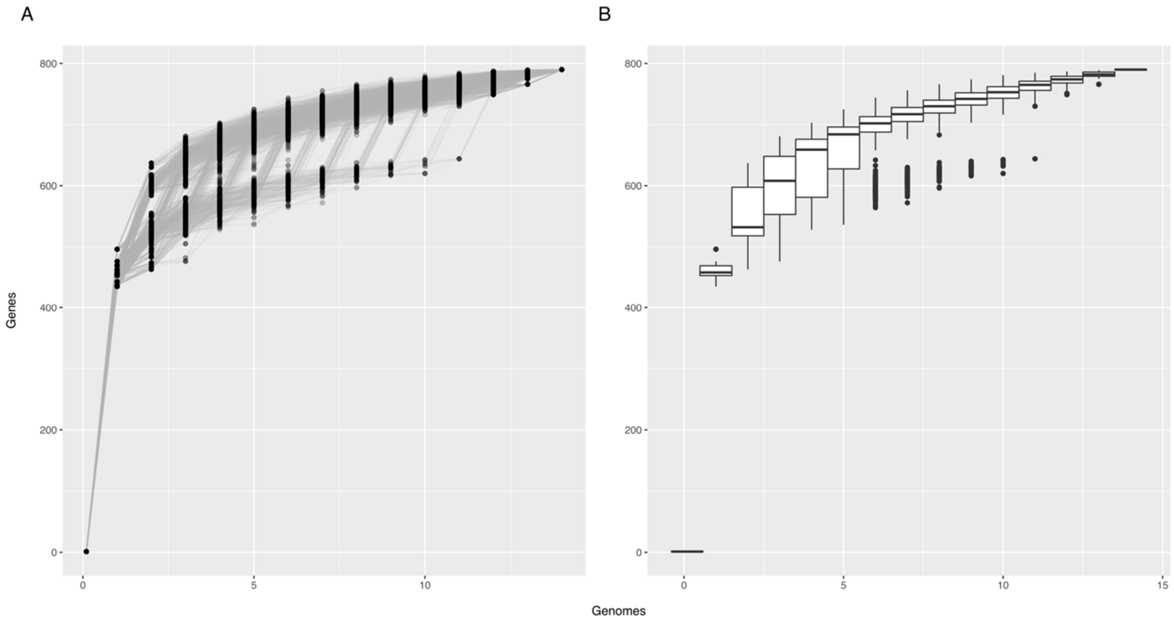


**Figure S4**. Rarefaction curve of Coccolithovirus strains. This rarefaction curve shows the number of genes present in the pangenome with varying numbers of genomes considered: (**A**) All 1000 permutations of the rarefaction curve are shown. (**B**) Boxplots show the overall range of each point of the curves.

A

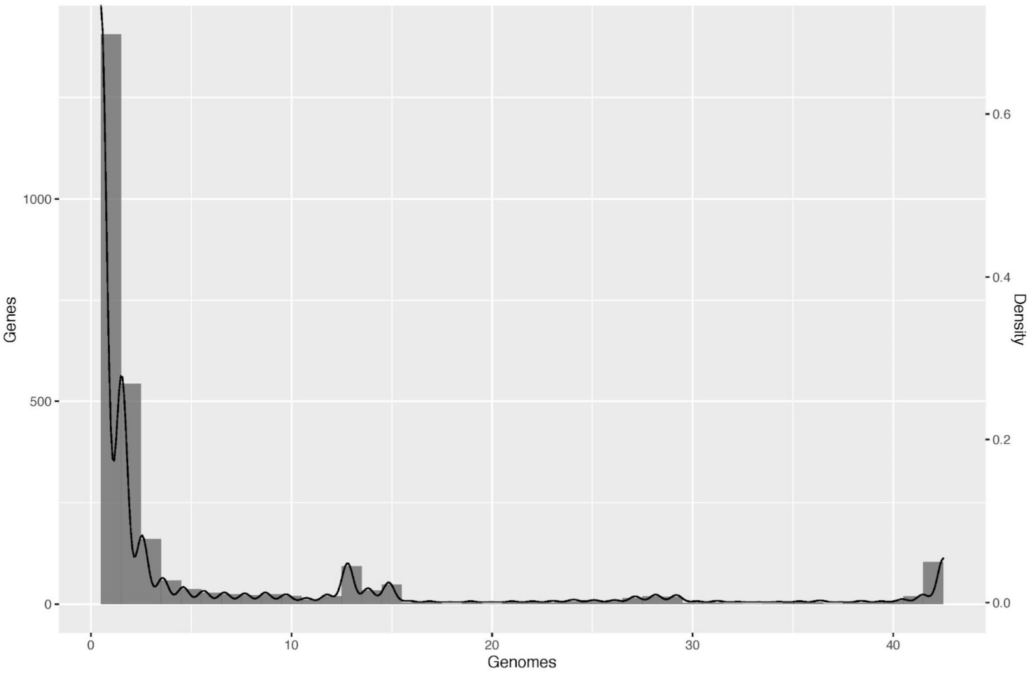


B

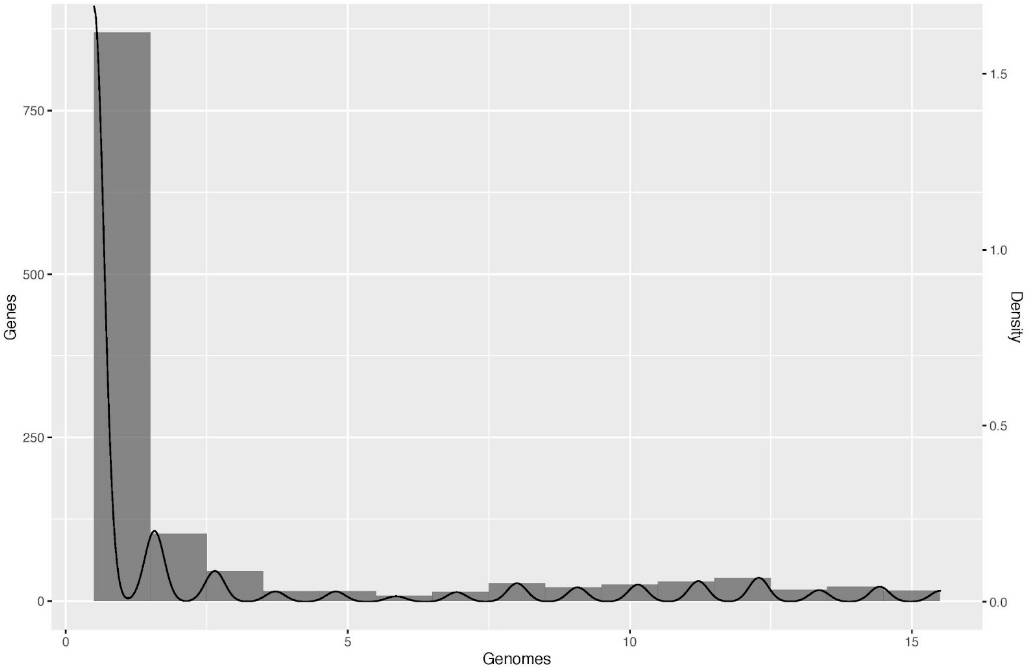


**Figure S5**. Pangenome distributions for Chloroviruses and Prasinoviruses: (**A**) Pangenome distribution across the 42 Chlorovirus genomes and (**B**) the 16 Prasinovirus genomes. A histogram of how many genomes a gene cluster is found in. An aligned relative density estimate of the histogram is displayed on the alternative y-axis (right).

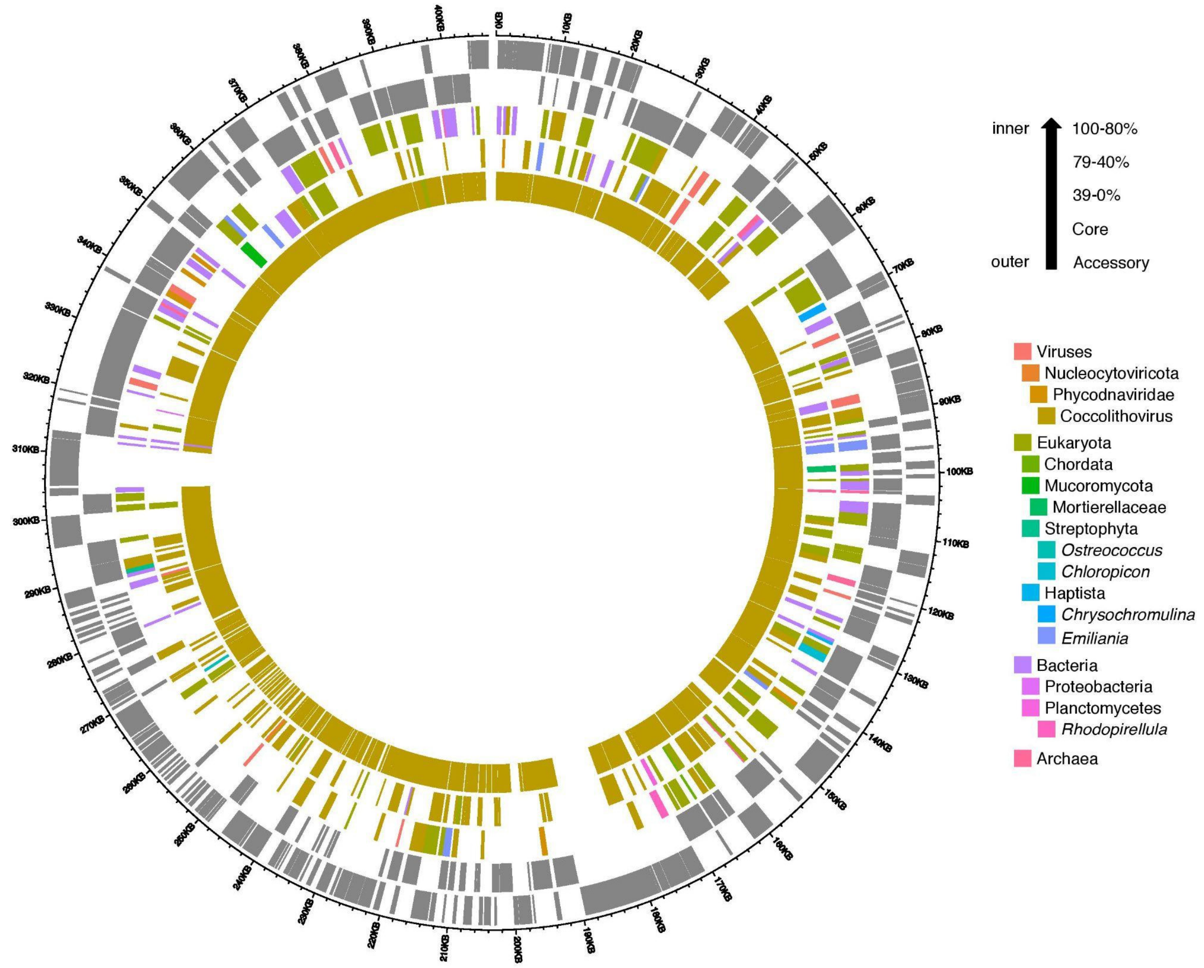


**Figure S6.** The majority taxonomy of EhV-86 BLASTP hits, divided into three alignment percent identity ranges. Visualized using the shinyCircos app https://yimingyu.shinyapps.io/shinycircos/ and the R package ComplexHeatmap v2.6.2. ehv060 (putative lectin protein), ehv192 (putative membrane protein), ehv204 (putative membrane protein), and ehv364 (putative membrane protein) are long and/or repetitive and did not have blast results to include here.
